# Supplementary material for: Can an Aerobic Exercise Programme Improve the Response of the Growth Hormone in Fibromyalgia Patients? A Randomised Controlled Trial
Source: Int J Environ Res Public Health. 2023 Jan 27;20(3):2261. doi: 10.3390/ijerph20032261 (PMC9915310; doi:10.3390/ijerph20032261)
Supplement: Supplementary file 1 [file ijerph-20-02261-s001.zip › ijerph-2168642-supplementary.pdf]

Supplement 1: Details of the training programme

The training program included integrated work on aerobic endurance and dynamic strength. The sessions were mainly based on low-impact aerobic dance (lower intensity, lower risk of injury, and less repercussions at the joint level than high-impact dance), combined with strength exercises with self-loads, and relaxation techniques for the 'back-to-the-calm' phase. The programme was adapted to the ACSM guidelines (Busch et al., 2007), with the sessions following the same structure:

1. Warm-up (10-15 minutes): Locomotion exercises, including joint mobility work, always respecting comfortable ranges of motion for each participant.

2. Main part (30-35 minutes):

2.1. Cardiovascular endurance work through low-impact aerobic dance, including mainly global and locomotion movements, involving both the lower and upper body.

2.2 Strength endurance work, through calisthenics, performing a total of 6 to 7 exercises, with 15 to 25 repetitions each, as established by the ACSM guidelines to improve muscular endurance (Pescatello, 2014). Exercises included squat, forward lunges, shoulder bridge, chest lift, superman, bicycle in air, top leg abduction, etc.

3. Back-to-the-calm (10-15 minutes): Relaxation techniques and breathing exercises.

The intensity of the sessions was controlled through pulsometers, registering an average heart rate in the aerobic part of around 140 BPM, which would correspond to values close to 80% of the Maximum Heart Rate, considering the mean age of programme participants for its establishment. As soon as the session finished, the Borg scale (Borg, 1976) was passed to verify if the training progression was adequate.

References

Borg G. Simple rating methods for estimation of perceived exertion. Wenner-Gren Center International Symposium 1976. Series 28:39-47.

Busch AJ, Barber KA, Overend TJ, et al. Exercise for treating fibromyalgia syndrome. Cochrane Database Syst Rev. 2007; CD003786.
doi: 10.1002/14651858.CD003786.pub2.

Pescatello L. In: ACSM's Guidelines for Exercise Testing and Prescription. 9th ed. Pescatello L.S., Arena R., Riebe D., Thompson P.D., editors. Volume 58. ASCM Group Publisher; Baltimor, MD, USA: Philadelphia, PA, USA: 2014. p. 328. The Journal of the Canadian Chiropractic Association.
